# The role of PSMA PET/CT in predicting downgrading in patients with Gleason score 4+4 prostate cancer in prostate biopsy

**DOI:** 10.1007/s00345-024-05012-2

**Published:** 2024-05-21

**Authors:** Ibrahim Can Aykanat, Yakup Kordan, Hulya Seymen, Ersin Koseoglu, Arif Ozkan, Baris Esen, Kayhan Tarim, Ibrahim Kulac, Okan Falay, Bengi Gurses, Dilek Ertoy Baydar, Abdullah Erdem Canda, Mevlana Derya Balbay, Mehmet Onur Demirkol, Tarik Esen

**Affiliations:** 1 Department of Urology, Koc University Hospital, Zeytinburnu, 34010 Istanbul, Turkey; 2 Department of Urology, Koc University School of Medicine, Istanbul, Turkey; 3 Department of Nuclear Medicine, Koc University School of Medicine, Istanbul, Turkey; 4 Department of Pathology, Koc University School of Medicine, Istanbul, Turkey; 5 Department of Radiology, Koc University School of Medicine, Istanbul, Turkey; 6 Rahmi M. Koc Academy of Interventional Medicine, Education and Simulation, RMK AIMES, Istanbul, Turkey; 7 Urology Clinic, VKF American Hospital, Istanbul, Turkey

**Keywords:** Prostate cancer, Downgrading, High risk, PSMA PET CT, Gleason score 8

## Abstract

**Background:**

To investigate the predictable parameters associated with downgrading in patients with a Gleason score (GS) 8 (4+4) in prostate biopsy after radical prostatectomy.

**Methods:**

We retrospectively analyzed 62 patients with a GS of 4+4 on prostate biopsy who underwent robotic radical prostatectomy between 2017 and 2022.

**Results:**

38 of 62 (61.2%) were downgraded. In multivariable logistic regression model, Ga-68 prostate-specific membrane antigen (PSMA) positron-emission tomography (PET)/computed tomography (CT) SUV max was independent predictor of downgrading (OR 0.904; *p* = 0.011) and a Logistic Regression model was constructed using the following formula: *Y* = 1.465–0.95 (PSMA PET/CT SUV max). The model using this variable correctly predicted the downgrading in 72.6% of patients. The AUC for PSMA PET/CT SUV max was 0.709 the cut off being 8.8.

A subgroup analysis was performed in 37 patients who had no other European Association of Urology (EAU) high risk features. 25 out of 37 (67.5%) were downgraded, and 21 of these 25 had organ confined disease. Low PSMA SUV max (<8.1) and percentage of GS 4+4 biopsy cores to cancer bearing cores (45.0%) were independently associated with downgrading to GS 7.

**Conclusion:**

PSMA PET/CT can be used to predict downgrading in patients with GS 4+4 PCa. Patients with GS 4+4 disease, but no other EAU high risk features, low percentage of GS 4+4 biopsy cores to cancer bearing cores, and a low PSMA PET/CT SUV max are associated with a high likelihood of the cancer reclassification to intermediate risk group.

**Supplementary Information:**

The online version contains supplementary material available at 10.1007/s00345-024-05012-2.

## Introduction

Prostate cancer (PCa) is the second most common cancer in men and the fifth leading cause of cancer-related death [1]. PCa diagnosed by prostate biopsy is classified by the Gleason scoring system. Risk classification is crucial in selecting the appropriate treatment and patients with a total Gleason score (GS) ≥ 8 in their prostate biopsy are classified as having high-risk disease according to the European Association of Urology (EAU) risk classification, regardless of other parameters [2]. Shared decision making in a high risk non-metastatic patient has some unique features like radical prostatectomy being possibly the first step of a multimodal treatment or duration and/or content of hormonal manipulation together with radiation treatment [2, 3]. Yet, the literature has shown that Gleason 4+4 PCa diagnosed by prostate biopsy is a very heterogeneous group, with a 40–60% rate of downgrading to GS ≤ 7 in final pathology after RP [4–11]. In patients with a GS 7 and no other high risk features (PSA < 20 and no cT3 findings) treatment details may considerably differ from the ones in high risk patients.

There are only a few studies in the literature investigating downgrading in high-risk PCa. None of them examined the role of Ga-68 Prostate-specific membrane antigen (PSMA) positron-emission tomography (PET)/computed tomography (CT) used as primary staging tool in these patients. With this study we aimed to investigate a comprehensive analysis of the downgrading dynamics in patients diagnosed with GS 8 (4+4) prostate cancer via biopsy, specifically focusing on the role of PSMA PET/CT as a primary staging tool to predict downgrading following radical prostatectomy.

## Materials and methods

After approval of Koç University Ethical Committee, we retrospectively reviewed the files of 633 patients who underwent robotic radical prostatectomy (RARP) at Koç University Hospital and VKV American Hospital between 2017 and 2022. The study was performed in accordance with the Declaration of Helsinki. Patients with at least one positive core of GS 4+4 in the prostate biopsy as their highest GS were included in the study. Patients who received neoadjuvant hormonal therapy/chemotherapy/radiotherapy or who had missing clinical data for prostate biopsy, multiparametric magnetic resonance imaging (mpMRI) of the prostate or Gallium 68 PSMA PET/CT were excluded.

12 cores were obtained either systematically or in the form of magnetic resonance imaging/transrectal ultrasound (MRI/TRUS) fusion-guided biopsy for each patient. Multiple biopsies taken from the target lesion are considered as a single positive core with the highest GS assigned. All biopsy and surgical pathology materials were centrally reviewed by the same uropathology team.

All patients were evaluated with age, preoperative prostate specific antigen (PSA), PSA density, mpMRI findings (prostate volume, Prostate Imaging-Reporting and Data System v 2.1 (PI-RADS) lesion score, target lesion size, clinical T stage), prostate biopsy parameters (positive cancer cores, percentage of positive cancer cores to total biopsy cores, positive Gleason 4+4 cancer cores, percentage of positive Gleason 4+4 cores to total biopsy cores, percentage of positive Gleason 4+4 cores to total number of cancer bearing cores, PSMA PET/CT findings (clinical stage, SUV max value of lesion). Clinical staging was determined by digital rectal examination, mpMRI and PSMA PET/CT findings, and the most advanced stage in the findings was accepted as the clinical stage of the patient.

### Statistics

For descriptive purposes, the mean, standard deviation (SD), median, interquartile range (IQR) where applicable were presented for continuous variables, while the number and percentage were presented for categorical variables. Continuous variables were tested using *t* test and Mann–Whitney *U* test and the categorical variables were tested using a chi-square test. The prediction of downgrading was performed using logistic regression. All potential predictors were first tested separately in univariable models. Stepwise logistic regression was then performed to select a set of independent statistically significant predictors in a multivariable model, allowing entrance of variables having *p* < 0.10 in the univariable regression. The results of logistic regression analyses are presented as odds ratios (ORs), 95% confidence intervals (CIs) and *p* values. The Hosmer and Lemeshow test was used to assess the goodness of fit of the model. In order to estimate the area under the curve (AUC) for predicting GS downgrade to determine the diagnostic performance of PSMA PET/CT SUV max value, a receiver operating characteristic (ROC) curve analysis was conducted. The AUC between 0.70 and <0.80 is interpreted as acceptable, 0.80 and <0.90 as excellent and ≥0.90 as outstanding [12]. All statistical tests were 2-sided with *p* < 0.05 considered to be statistically significant. Analyses were conducted using SPSS 22.0 (Chicago, Illinois) software.

## Results

A total of 62 patients were included for analysis. In the pathological examinations after RARP, 12 (19.3%) patients’ GS were downgraded to Gleason 3+4, 26 (41.9%) patients were downgraded to Gleason 4+3, 11 (17.7%) patients GSs were not change and 13 (20.9%) patients GSs were upgraded.

The preoperative and postoperative data of the patients and the comparison between the groups were given in Table 1. SUV max of the intraprostatic lesion in PSMA PET/CT was statistically lower in downgraded patients compared to real GS 4+4 patients (*p* = 0.006). In prostate biopsy parameters, except for the ratio of Gleason 4+4 cores to cancer bearing cores (*p* = 0.010), there were no statistically differences between the groups. Regarding pathologic outcomes, there was no statistical differences between groups in pathological T stage and surgical margin status after radical prostatectomy (*p* = 0.237, 0.306, respectively). However, patients with downgraded disease were significantly less likely to have lymph node metastasis (*p* = 0.023) (Table 1).

**Table 1:** Patient characteristics associated with downgrading to Gleason score 7

	Total (*n* = 62)	Downgrade (*n* = 38)	No downgrade (*n* = 24)	*p*
Age (years)				0.553
Mean (SD)	64.3 (7.3)	64.7 (7.8)	63.5 (6.6)	
Median (range)	66.0 (59.0–70.0)	66.0 (60.0–70.2)	65 (58.2–69.5)	
PSA (ng/ml)				0.594
Mean (SD)	10.7 (11.9)	8.5 (2.4)	9.6 (4.9)	
Median (range)	8.1 (5.4–11.5)	7.9 (5.4–11.2)	9.1 (5.2–12.6)	
PSA density				0.988
Mean (SD)	0.29 (0.49)	0.33 (0.62	0.21 (0.10)	
Median (range)	0.20 (0.13–0.27)	0.19 (0.12–0.28)	0.20 (0.14–0.27)	
Prostate volume				0.272
Mean (SD)	46.0 (21.0)	44.7 (24.4)	48.1 (14.1)	
Median (range)	41.5 (32.4–55.0)	39 (28.7–50.5)	48 (40.0–57.2)	
Digital rectal examination stage (*n*) (%)				0.336
1	17 (27.4)	8 (21.1)	9 (37.5)	
2	29 (46.8)	20 (52.6)	9 (37.5)	
3	16 (25.8)	10 (26.3)	6 (25.0)	
Lesion size (mm)				0.275
Mean (SD)	13.7 (8.0)	12.6 (7.4)	15.3 (8.9)	
Median (range)	12.7 (7.3–18.0)	11.7 (7.0–18.0)	13 (10–21.2)	
PIRADS lesion (*n*)(%)				0.842
2	1 (1.6)	1 (2.6)	0 (0)	
3	3 (4.8)	2 (5.3)	1 (4.2)	
4	37 (59.7)	23 (60.5)	14 (58.3)	
5	21 (33.9)	12 (31.6)	9 (37.5)	
mpMRI stage (*n*) (%)				0.291
T2	37 (59.7)	25 (65.8)	12 (50.0)	
T3a	15 (24.2)	8 (21.1)	7 (29.2)	
T3b	8 (12.9)	5 (13.2)	3 (12.5)	
T4	2 (3.2)	0 (0)	2 (8.3)	
PSMA PET/CT Stage (*n*) (%)				0.220
Prostate localize	54 (87.1)	36 (94.7)	18 (75.0)	
LN inv	7 (14.5)	2 (5.3)	5 (20.8)	
Metastasis	1 (1.6)	0	1 (4.2)	
PSMA PET/CT SUV max value				0.006*
Mean (SD)	10.7 (9.5)	8.0 (6.6)	14.8 (11.9)	
Median (range)	6.9 (4.6–14.6)	6.1 (4.3–8.1)	12.9 (6–19.9)	
Biopsy type (*n*) (%)				0.297
Systematic biopsies	31 (50)	21 (55.3)	10 (41.7)	
MRI/TRUS fusion-guided biopsies	31 (50)	17 (44.7)	14 (58.3)	
Positive cancer cores (*n*)				0.760
Mean (SD)	6.1 (3.0)	6.0 (3.0)	6.1 (3.0)	
Median (range)	6.0 (4.0–8.0)	6 (4.0–8.0)	7.0 (2.5–8.7)	
Percent of positive cancer cores out of all biopsy cores (%)				0.578
Mean (SD)	45.6 (22.5)	45.1 (22.3)	46.5 (23.4)	
Median (range)	46.1 (30.7–61.5)	44.5 (30.7–60.3)	50.0 (22.8–66.6)	
Positive GS 4+4 biopsy cores (*n*)				0.217
Mean (SD)	3.4 (2.6)	3.0 (2.9)	3.9 (2.0)	
Median (range)	2.0 (1.0–5.0)	2.0 (1.0–3.2)	4 (2.0–6.0)	
Percentage of positive GS 4+4 biopsy cores out of all biopsy cores (%)				0.228
Mean (SD)	25.6 (20.9)	23.0 (23.4)	29.6 (15.5)	
Median (range)	16.6 (8.3–36.1)	15.3 (7.6–25.5)	30 (16.6–41.2)	
Percentage of GS 4+4 cores to cancer bearing cores (%)				0.010*
Mean (SD)	59.0 (31.0)	51.1 (30.9)	71.6 (27.3)	
Median (range)	50 (33.3–100.0)	40 (25.0–78.5)	70.8 (51.0–100)	
Percentage of GS 4+4 per core (%)				0.168
Mean (SD)	61.4 (28.4)	57.8 (27.0)	66.9 (30.3)	
Median (range)	66.0 (38.7–88.5)	57.5 (35.0–80.0)	70 (50.0–90.0)	
Radical prostatectomy pathology stage (*n*) (%)				0.433
T2	32 (51.6)	22 (57.9)	10 (41.7)	0.237
T3a	16 (25.8)	9 (23.7)	7 (29.2)	0.275
T3b	14 (22.6)	7 (18.4)	7 (29.2)	0.678
Lymph node involvement (*n*) (%)				0.023**
Positive	16 (25.8)	6 (15.8)	10 (41.7)	
Negative	46 (74.2)	32 (84.2)	14 (58.3)	
Surgical margin (*n*) (%)				0.306
Positive	26 (41.9)	14 (36.8)	12 (50.0)	
Negative	36 (58.1)	24 (63.2)	12 (50.0)	

*Statistically significant, Mann whitney *U*

**Statistically significant, Chi-square

It is observed that the significance values in the table are in bold

**Fig. 1:**
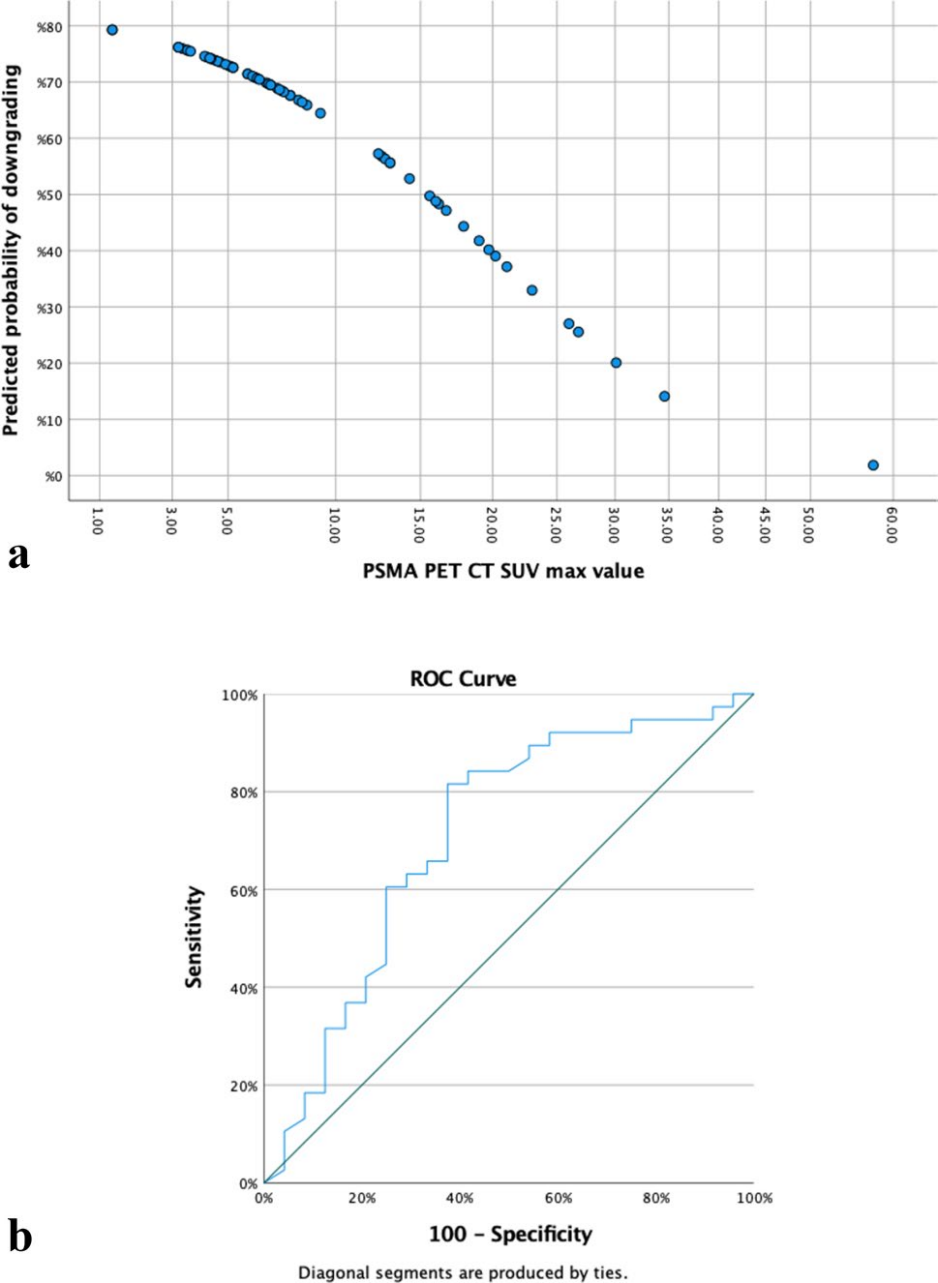
**a** A scatterplot graph created by the model. **b** Receiver operating characteristic (ROC) curve analyses were conducted to evaluate the performance of PSMA PET CT SUV max in predicting pathologic downgrade

In uni- and multivariable logistic regression models predicting downgrading in GS 4+4 patients, PSMA SUV max was a modest, independent predictor of downgrading (multivariable OR 0.904; 95% CI 0.836–0.977; *p* = 0.011). Other covariates failed to reach independent predictor status downgrading in GS 4+4 (Table 2). According to their respective coefficients, the Logistic Regression (LR) model was constructed using the following formula: *Y* = 1.465–0.95 (PSMA SUV max value) (Fig. 1a). The model with this variable correctly predicted the downgrading in 72.6% of patients. AUC for PSMA SUV max was 0.709 (95% CI 0.569–0.849) (Fig. 1b). The cut off value which was determined by Youden index for PSMA SUV max was 8.8, with sensitivity, specificity, positive predictive value and negative predictive value of 81.5, 62.5, 77.5, 68.2%, respectively. Positive likelihood ratio was 2.17, negative likelihood ratio was 0.296.

**Table 2:** Uni- and multivariate logistic regression analyses were conducted to identify factors associated with downgrading to Gleason 7 for patients with Gleason 4+4 prostate cancer in prostate biopsy

	Univariate		Multivariate	
	OR 95% CI	*p*	OR 95% CI	*p*
PSA (ng/ml)	1.014 (0.962–1.069)	0.600		
PSA density	3.355 (0.191–58.878)	0.408		
Lesion size (mm)	0.959 (0.899–1.023)	0.200		
Digital rectal examination stage	0.714 (0.350–1.453)	0.353		
mpMRI Stage	1.136 (0.946–1.365)	0.173		
PSMA PET/CT SUV max	0.910 (0.843–0.981)	0.015*	0.904 (0.836–0.977)	0.011*
Positive cancer cores (*n*)	0.987 (0.833–1.171)	0.884		
Percentage of positive cancer cores out of all biopsy cores (%)	0.985 (0.961–1.010)	0.233		
Positive GS 4+4 biopsy core (*n*)	0.885 (0.729–1.076)	0.220		
Percentage of positive GS 4+4 biopsy cores out of all biopsy cores (%)	0.985 (0.961–1.010)	0.233		
Percentage of GS 4+4 cores to cancer bearing cores (%)	0.978 (0.960–0.995)	0.013*		
Percentage of positive GS 4+4 core ratio	0.988 (0.970–1.007)	0.221		

*Statistically significant

We performed a subgroup analysis to investigate the factors to predict downgrading in patients with Gleason 4+4 PCa with no other EAU high risk features (PSA < 20 ng/ml and cT2 stage and without extracapsular extension (ECE) suspicion on either mpMRI or PSMA PET-CT). A total of 37 patients met the inclusion criteria. 25 of 37 (67.5%) were downgraded (9/25 downgraded to Gleason 3+4, 16/25 downgraded to Gleason 4+3). Low PSMA SUV max and low percent of Gleason 4+4 biopsy cores to tumor bearing cores were independently associated with downgrading to Gleason score 7 in our multivariate analyses (Table 3). A cutoff value of 8.1 was calculated with an AUC of 73.5% for PSMA SUV max, and a cutoff value of 45.0% was determined with an AUC of 73.5% for the percentage of Gleason 4+4 biopsy cores to tumor bearing cores. A total of 4 patients out of 25 who were downgraded, were still considered to have high-risk disease as they were diagnosed with locally advanced prostate cancer in final pathological examination. The remaining 21 (56.7%) patients were thus reclassified into the intermediate-risk category.

**Table 3:** Uni- and multivariate logistic regression analyses were conducted to identify factors associated with downgrading to Gleason 7 for patients with Gleason 4+4 prostate cancer in prostate biopsy with no other EAU high risk features

	Univariate		Multivariate	
	OR 95% CI	*p*	OR 95% CI	*p*
PSA (ng/ml)	0.926 (0.757–1.132)	0.453		
PSA density	5.977 (0.009–3895.116)	0.589		
Lesion size (mm)	0.996 (0.892–1.111)	0.939		
PSMA PET/CT SUV max value	0.862 (0.757–0.981)	0.024*	0.843 (0.736–0.966)	0.014*
Positive cancer cores (*n*)	0.936 (0.744–1.177)	0.570		
Percentage of positive cancer cores out of all biopsy cores (%)	0.988 (0.957–1.021)	0.478		
Positive GS 4+4 biopsy core (*n*)	0.684 (0.467–1.002)	0.051		
Percentage of positive GS 4+4 biopsy cores out of all biopsy cores (%)	0.943 (0.892–0.996)	0.037*		
Percentage of GS 4+4 cores to cancer bearing cores (%)	0.971 (0.945–0.998)	0.033*	0.964 (0.934–0.995)	0.023*
Percentage of positive GS 4+4 core ratio	0.992 (0.969–1.017)	0.540		

*Statistically significant

## Discussion

In this retrospective analysis of 62 patients diagnosed with GS 8 (4+4) prostate cancer, we discovered a downgrading rate of 61.2% post-radical prostatectomy, aligning with reported literature trends [4–9, 11]. In a disease with such a high downgrading rate, the risk classification and consequently the treatment plan may change. Therefore, it is crucial to identify parameters that can predict patients whose disease most probably will be downgraded.

Qi et al. observed that downgrading was associated with a lower PSA, a lower number of Gleason 8 biopsy cores, the presence of Gleason pattern 3 on biopsy cores, and a smaller percentage of tumor. Additionally, they found that patients with pT2 stage, without positive surgical margins, or without extracapsular extension tended to be downgraded [5]. Ginsburg et al. observed that having ≤2 cores with Gleason score 8, ≤50% maximal tumor involvement of any individual core positive for Gleason score 8, or the presence of Gleason pattern 3 in other biopsy cores were associated with downgrading in their multivariable model [6]. Altok et al. showed that a low number of GS 4+4 biopsy cores was associated with downgrading in univariate analyses, but not in multivariate analysis [8]. Ranasingle et al. showed that lower prostate specific antigen, fewer positive biopsy cores and lower clinical stage were associated with downgrading [7]. Wenzel et al. examined the characteristics of 6690 patients with high-risk prostate cancer from the NCCN database and they developed a model that predicted downgrading using the parameters of biopsy Gleason patterns, PSA, cT stage, and the number of positive biopsy cores. Their nomogram had an accuracy of 71.0% in predicting any downgrading [9].

Unlike the above studies, we did not detect a statistical relationship between PSA value and the probability of downgrading. Although the percentage of GS 4+4 cores to cancer bearing cores were statistically lower in downgraded patients comparing to grade-stable patients, it did not reach to statistical significance in multivariate analyses. Furthermore, we did not observe a statistically significant difference between patients with or without downgrading in terms of clinical or pathological T stage. We found that the SUV max of the lesion in PSMA PET/CT was the only parameter that predicted downgrade in multivariate analyses.

In the treatment of high-risk PCa, the cancer stage, along with GS affects the treatment decision. MpMRI and PSMA PET/CT are currently used diagnostic tools for local and distant staging [13]. When both tests are used together, the ability to predict extracapsular extension is increased [14, 15]. In our study, we found that PSMA PET/CT is not only useful for staging purposes but can also be a predictor of the final pathological GS in PCa. In patients with clinically localized PCa, a low PSMA PET/CT SUV max and a low percentage of GS 4+4 cores to cancer bearing cores predict an increased likelihood of downgrading and thus reclassification to intermediate-risk disease according to EAU and NCCN guidelines. This may affect the details of the preoperative shared decision making in terms of treatment choice in general, duration of hormonal manipulation together with radiation treatment, even the extent of a safe nerve sparing surgery. In our study 21 out of 37 Gleason 4+4 PCa patients (56.7%) with a PSA < 20 and clinically organ confined disease were reclassified as intermediate risk group. To our knowledge, this is the first study reporting the collateral ability of PSMA PET/CT in predicting downgrading for biopsy GS 4+4 patients following radical prostatectomy.

While our study benefits from a thorough central review of PSMA PET/CT scans and biopsy specimens by a specialized expert team, it is not without limitations. The retrospective nature of our analysis introduces potential biases inherent in such studies. Moreover, the relatively small sample size compared to previous multicenter studies may limit the generalizability of our findings.

## Conclusions

PSMA PET/CT can be a tool to predict downgrading in patients with GS 4+4 PCa. For patients with biopsy GS 4+4 PCa and no other EAU high risk features, there is a high likelihood of risk reclassification if they have low Percentage of GS 4+4 cores to cancer bearing cores and a low PSMA PET/CT SUV max. To be able to predict pathological downgrading in patients with biopsy GS 4+4 PCa may help the clinician to have a more detailed and structured presentation of the treatment options to the patient.

## Supplementary Information

Below is the link to the electronic supplementary material.

Supplementary file1 (DOCX 17 KB)

## Data Availability

The data used in this study were collected by our research team and are not publicly available due to ethical requirements to protect participant confidentiality. Access to the data can be provided under limited conditions after obtaining the appropriate ethical approval. Researchers interested in accessing the data should contact (Corresponding author: Ibrahim Can Aykanat, e-mail: canaykanat89@gmail.com)
