# Supplementary material for: The role of PSMA PET/CT in predicting downgrading in patients with Gleason score 4+4 prostate cancer in prostate biopsy
Source: World J Urol. 2024 May 21;42(1):341. doi: 10.1007/s00345-024-05012-2 (PMC11108863; doi:10.1007/s00345-024-05012-2)
Supplement: Supplementary file 1 — Supplementary file1 (DOCX 17 KB) [file 345_2024_5012_MOESM1_ESM.docx]

**Revision Note**

Dear Editor,

We have revised our manuscript entitled “ **The Role of PSMA PET/CT in Predicting Downgrading in Patients with Gleason Score 4+4 Prostate Cancer in Prostate Biopsy” (Manuscript # WJUR-D-23-01782).** In the revised manuscript, all revisions are shown in highlighted characters. Our responses to reviewers comments are as follows:

Respond to reviewer 1

***Q1. Abstract is confusing. Please re-write it in simple manner. First in methods the authors wrote 633 patients treated with RARP then out of knowhere 62 were included. I stronlgy suggest to to write that you included 62 patients with GS 4+4.***

**A1.** Thank you very much for your contribution. In line with the reviewer's suggestion, we have revised the methods section of our abstract to read as follows: 'We retrospectively analyzed 62 patients with a Gleason Score (GS) of 4+4 on prostate biopsy who underwent robotic radical prostatectomy between 2017 and 2022.”

***Q2. Did all the patients undergo MRI targeted biopsies? Very high rates of downgrading in authors cohort. There is something wrong in Table 1 with percentage. I suggest to put % vertically (and calculated), separately for downgrading and no downgrading - for example 9 pts with PIRADS 5 is 37.5% of all patients in this group etc. please do it to all otherwise it is hard to analyze. How about DRE data? As for now DRE should be used for local staging, not mpMRI.***

A2. Thank you very much for your question. In response to the reviewer's question, our patients' prostate biopsies were taken either systematically or as magnetic resonance imaging/transrectal ultrasound (MRI/TRUS) fusion-guided biopsies. To avoid any confusion, we have changed the term 'MR-guided fusion biopsy' in the Materials and Methods section to 'magnetic resonance imaging/transrectal ultrasound (MRI/TRUS) fusion-guided biopsies.' Additionally, the information on the type of biopsy for each group has been added to Table 1. As the reviewer pointed out, it appears that our downgrade rates are high; however, the downgrade rates in the literature are similar. Downgrade rates are observed to be 59% in Ginsburg et al., 45% in Ranasighne et al., 50.5% in Altok et al., and 60.1% in Wenzel et al. Based on your suggestions, we have recalculated the percentage values of the figures given in Table 1. Also, we have added our Digital Rectal Examination (DRE) findings to Tables 1, 2, and 3.

***Q3.  the OR is calculated per 1 unit of SUVmax? Please clarify. The same apply to percentage of GS 4+4.***

We thank the reviewer for their valuable feedback. In Table 2, it is observed that each unit increase in SUVmax in PSMA PET/CT decreases the probability of downgrade by 9%. Since the SUVmax value of PSMA PET/CT does not have a unit, it could not be specified. In Table 3, it is shown that each unit increase in SUVmax in PSMA PET/CT decreases the probability of downgrade by 15.7%, and each unit increase in the percentage of GS 4+4 cores to cancer-bearing cores decreases the probability of downgrade by 3.6%

**Reviewer #2**

***Q1. The aim is provided at the end of the Introduction section, yet I would suggest re-phrasing and expanding it (e.g., that used in the abstract is more properly stated).***

A1. We thank the reviewer for their evaluation. In accordance with their suggestions, we have expanded and rewritten the aim section in the last sentence of the introduction. The sentence "With this study we aimed to investigate downgrading dynamics in patients with GS 8 (4+4) in prostate biopsy by using PSMA PET/CT." has been changed to "With this study we aimed to investigate a comprehensive analysis of the downgrading dynamics in patients diagnosed with GS 8 (4+4) prostate cancer via biopsy, specifically focusing on the role of PSMA PET/CT as a primary staging tool to predict downgrading following radical prostatectomy."

***Q2. Discussion is adequately presented, and interpretations are well stated and justified by results. However, I would suggest expanding the first sentence, including the main characteristic of the study in terms of setting and population (i.e., not only stating "in our study, ..", yet e.g., ".. patients with GS 4+4 …). Similarly, Limitations and strengths of the study could be more properly acknowledged/discussed.***

A2. The sentence “In our study, we found that 61.2% of the patients PCa was downgraded, which is consistent with the literature” has been changed to “In this retrospective analysis of 62 patients diagnosed with GS 8 (4+4) prostate cancer, we discovered a downgrading rate of 61.2% post-radical prostatectomy, aligning with reported literature trends”

Following the reviewer's suggestions, the sentence 'Despite several strengths, our study has certain limitations. First and foremost is the retrospective design of the study. Second, unlike previous retrospective studies based on large multicenter databases reported in the literature, this study was conducted with a smaller number of patients. Central review of the PSMA PET scans and the biopsy and radical pathology specimens by the same expert team are possible strengths.' has been changed to 'While our study benefits from a thorough central review of PSMA PET/CT scans and biopsy specimens by a specialized expert team, it is not without limitations. The retrospective nature of our analysis introduces potential biases inherent in such studies. Moreover, the relatively small sample size compared to previous multicenter studies may limit the generalizability of our findings.”

My highest Regards
